# Searching for a Common Thrombo-Inflammatory Basis in Patients With Deep Vein Thrombosis or Peripheral Artery Disease

**DOI:** 10.3389/fcvm.2019.00033

**Published:** 2019-04-02

**Authors:** Bram M. M. Kremers, Simone Birocchi, Rene van Oerle, Sacha Zeerleder, Henri M. H. Spronk, Barend M. E. Mees, Brenda M. Luken, Hugo ten Cate, Arina J. ten Cate-Hoek

**Affiliations:** ^1^Laboratory for Clinical Thrombosis and Hemostasis, Maastricht, Netherlands; ^2^Ospedale San Paolo, Milan, Italy; ^3^Department of Hematology and Central Hematology Laboratory, Inselspital, Bern University Hospital, University of Bern, Bern, Switzerland; ^4^Department for BioMedical Research, University of Bern, Bern, Switzerland; ^5^Immunopathology, Sanquin Research, Amsterdam, Netherlands; ^6^Amsterdam Infection and Immunity Institute, Amsterdam UMC, Amsterdam, Netherlands; ^7^Department of Vascular Surgery, Maastricht University Medical Center, Maastricht, Netherlands; ^8^Thrombosis Expertise Center, Maastricht, Netherlands; ^9^Department of Internal Medicine, Maastricht University Medical Center, Maastricht, Netherlands

**Keywords:** atherothrombosis, cardiovascular disease, coagulation, deep vein thrombosis, inflammation, neutrophil extracellular traps, peripheral artery disease

## Abstract

**Background:** Inflammation and hypercoagulability play a pivotal role in venous thromboembolism and atherothrombosis. Since venous thrombosis increases the risk of atherothrombotic events and vice versa, common mechanisms may be involved.

**Objectives:** To elucidate the role of neutrophils and coagulation in the occurrence of atherothrombotic events in patients with a history of deep vein thrombosis (DVT or peripheral artery disease (PAD).

**Materials and Methods:** We studied 115 patients from two cohorts (75 DVT, 40 PAD). From those with PAD, 20 patients had progressive disease; from those with DVT, 25 patients had a recurrent DVT and 25 suffered from post thrombotic syndrome (PTS); patients were age and sex matched to DVT and PAD patients without events. Markers of neutrophil recruitment (p-selectin) and activation [nucleosomes, human neutrophil elastase- α1anti-trypsin (HNE-AT)], an anti-inflammatory marker (Lipoxin A4) and a clotting activity marker (d-dimer), were measured with ELISA. Coagulation potential was analyzed by thrombin generation (CAT method).

**Results:** Higher nucleosome levels were found in DVT patients [11.3 U/mL (7.4–17.7)] compared to PAD patients [7.1 U/mL (5.1–13.8)], lower HNE-AT levels were found in DVT patients [33.4 ng/mL (23.5–40.5)] in comparison to PAD patients [158 ng/mL (88.1–283)]. No difference in nucleosome levels was found between DVT patients with cardiovascular (CV) events [12.6 U/mL (8.2–16.1)], and PAD patients with CV events [6.9 U/mL (4.9–11.2)]. Lipoxin A4 levels appeared to be significantly lower in DVT [2.4 ng/mL (1.7–4.8)] vs. PAD [35.6 ng/mL (16.6–80.1)], with similar results in DVT patients with CV events vs. PAD patients with CV events. Thrombin generation showed higher ETP [160.4% (141.1–215.4)], and peak height [292.1% (177.9–330)] values in DVT patients. D-dimer levels were significantly lower in the DVT cohort [330 ng/mL (220–550)] compared to the PAD cohort [550 ng/mL (369–959)].

**Conclusion:** In DVT patients, neutrophil activity does not appear to be an important driver of CV events. Although neutrophil activity is more pronounced in PAD, its effect is partly dampened by Lipoxin A4. Moreover, no associations were found between NET products and coagulation activity, suggesting that neutrophil activation does not play a pivotal role in the risk of thrombosis in either DVT or PAD.

## Introduction

There is an increased risk for atherothrombosis following deep venous thrombosis (DVT) (1–3). Prandoni et al. showed that the prevalence of symptomatic atherosclerotic plaques is significantly higher in patients with a history of DVT, suggesting there might be an overlap in the pathogenesis of both diseases (4). Moreover, epidemiological studies have highlighted the association between venous and arterial thrombosis, mainly focusing on the presence of common risk factors including obesity, diabetes, and hypertriglyceridemia (5). Although the increased risk of cardiovascular events following venous thrombosis has consistently been reported (6, 7), the opposite association is much less defined. Some authors report an increased risk of venous thromboembolism (VTE) in the first 3 to 6 months after an acute arterial cardiovascular event, but after that period, the increased risk disappears (8, 9). Common pathophysiological mechanisms linking arterial and venous thrombosis are most likely related to inflammation and hypercoagulability. Both arterial thrombosis, and to a lesser extent venous thrombosis, have been associated with increased markers of inflammation (10, 11). Additionally, increased thrombin generation *in vivo* (12) and *ex vivo* (13) has been associated with coronary artery disease as well as peripheral artery disease (PAD) and is also related to increased VTE occurrence (14). One mechanism linking inflammation and coagulation is the activation of neutrophils and the ability to adhere to platelets and endothelial surfaces. These cellular interactions are likely to contribute to a “prethrombotic state” or overt hypercoagulability. P-selectin is a cell adhesion molecule expressed by activated endothelium and activated platelets; its role in inflammation lies in the recruitment of neutrophils. Upregulation of p-selectin expression was shown to be associated with progression of atherosclerotic lesions (15) while inhibition resulted in accelerated thrombolysis and restoration of blood flow in thrombosed veins in animal studies (16, 17). Activation of neutrophils induces the formation of neutrophil extracellular traps (NETs), an important mechanism in the interplay between inflammation and coagulation. NETs are webs of de-condensed chromatin in the extracellular space with citrullinated histones and proteases, produced by activated neutrophils. NETs have thus far been described in relation to their antimicrobial activity (18), a scaffold function for red blood cells and platelets (19), and activation of platelets (20), facilitating the onset of thrombosis with increased thrombin generation (TG) (21–24).

Investigating the inflammatory and hypercoagulable role of NETs can be challenging because several markers are non-specific for NET formation. Extracellular nucleosomes for example, are not only released during NET formation, but levels can also be elevated due to necrosis and apoptosis (25). Unlike nucleosomes, human neutrophil elastase α1anti-trypsin (HNE-AT) complexes are more specific for the formation of NETs (26). HNE is known to cleave tissue factor pathway inhibitor (TFPI) and stimulate fibrin formation (16). Lipoxin, a metabolite of arachidonic acid, is a product of the leukocyte-platelet interaction with an important role in dampening the inflammatory response. The role of lipoxins in ischemia reperfusion is well-known (27), so one could speculate that lipoxin could also be protective in PAD and DVT.

Our aim with this explorative study, is to assess platelet activation and neutrophil recruitment as well as anti-inflammatory markers and markers of coagulation in patients with non-acute DVT and patients with peripheral artery disease (PAD), in order to elucidate the role of neutrophils and substantiate arguments for a common biochemical background for the occurrence of atherothrombotic events in these patient populations with chronic vascular disease.

## Materials and Methods

### Patients and Controls

Plasma was collected from patients from two previously established cohorts (PAD and DVT) at the Maastricht University Medical Center+ (MUMC+) (28, 29). From the cohort of patients with PAD, 40 patients were selected; 20 patients who experienced a cardiovascular (CV) event during follow-up and 20 patients who did not experience such an event, matched for age and sex to patients with CV events. Cardiovascular events were defined as myocardial infarction or angina pectoris (*n* = 1), stroke (*n* = 1), acute limb ischemia or revascularization procedure (*n* = 17), or the need for interventional treatment (*n* = 1).

**Figure 1:**
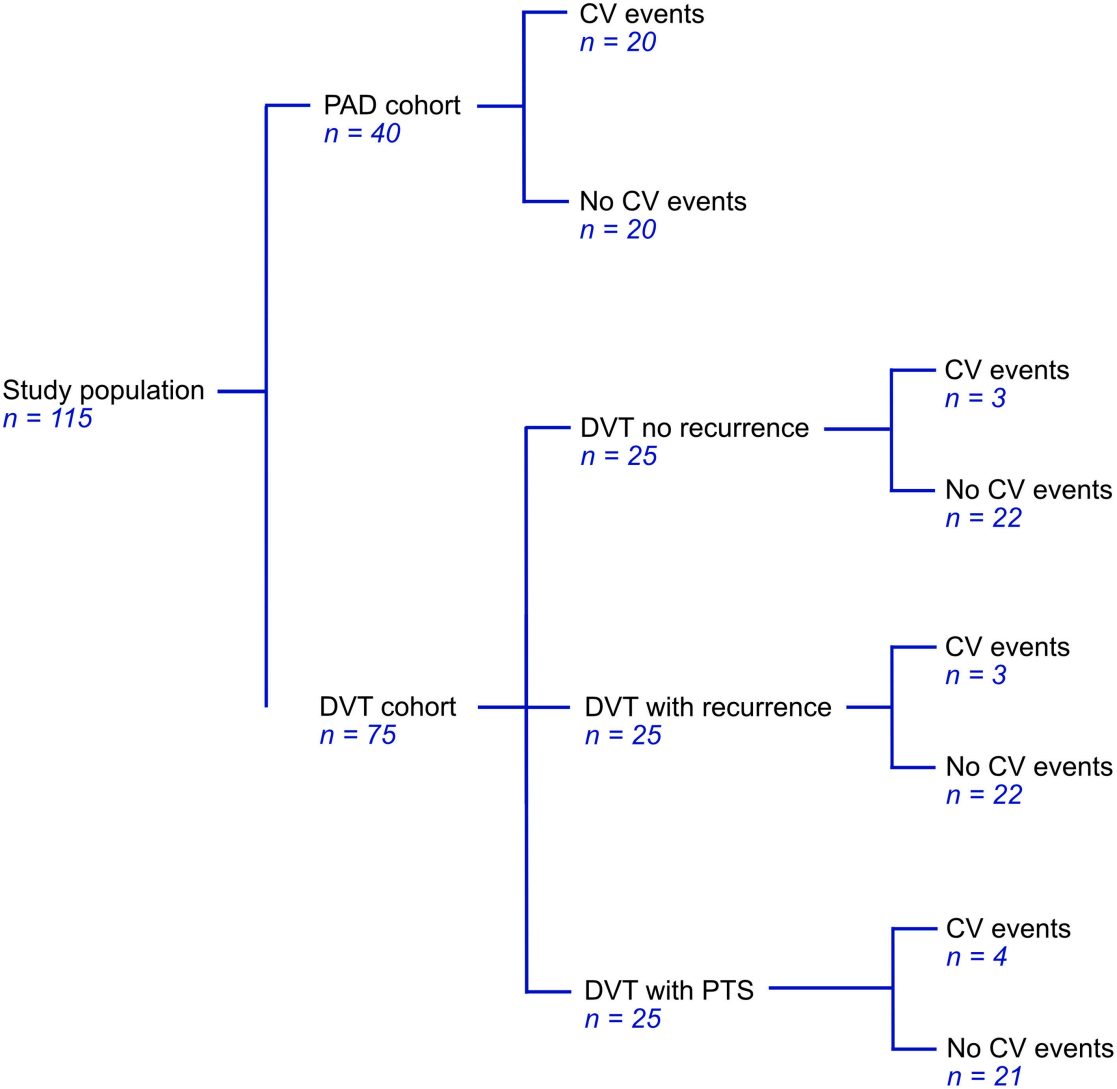
Cohort distribution of 40 patients with PAD and 75 patients with DVT.

Patients from the PAD cohort were newly diagnosed by ankle-brachial index (ABI) measurements, an ABI < 0.9 was considered to indicate PAD (30). Most of these patients were classified as stage IIa or IIb in the Fontaine classification (31). Patients were excluded based on the use of medication affecting coagulation (except for platelet aggregation inhibitors), documented congenital coagulation disorders, documented chronic inflammatory diseases, active malignancy, pregnancy, and age <18. Patients with DVT were selected from a cohort of patients that experienced at least one DVT. From this cohort, we selected a total of 75 patients; 25 patients who had experienced a recurrent event during follow-up, 25 age and sex matched patients that did not experience a recurrent thrombotic event and 25 patients from the same cohort that developed post-thrombotic syndrome (PTS) (Figure 1). Patients with documented chronic inflammatory diseases or with known venous insufficiency were excluded.

The Medical Ethics Committee (METC) of the Maastricht University Medical Center (MUMC+) approved both the PAD study (NL19929.068.07) and the DVT study (METC 15-4-256).

### Blood Collection and Storage

Venous blood was drawn from subjects in resting condition and collected by antecubital venipuncture with 21-gauge needles and 3.2% (w/v) citrated Vacutainer glass tubes. The tubes were processed using the standard platelet-poor plasma (PPP) centrifugation protocol used at our laboratory (2,000 × *g* for 5 min, 10,000 × *g* for 10 min). Samples were frozen and stored at −80°. Analysis was performed at one point in time, avoiding repeated freeze-thaw cycles.

### Measurements

Plasma levels of extracellular nucleosomes and HNE-AT were assayed using in-house ELISAs as described previously (32). To detect extracellular nucleosomes, monoclonal antibody CLB-ANA/60 (Sanquin, Amsterdam, the Netherlands) which recognizes histone 3 was coated. Biotinylated F(ab)2 fragments of monoclonal antibody CLB-ANA/58 (Sanquin, Amsterdam, the Netherlands), that recognizes the histone 2A/2B complex when bound to DNA was used for detection. As a standard, culture supernatant of apoptotic Jurkat cells

(1 × 10^6^ cells/mL) was used. One unit is the amount of nucleosomes released by ≈100 Jurkat cells. The lower detection limit of the assay was 2.5 U/mL, the coefficients of variation were 8.5% (inter-assay) and 4.3% (intra-assay).

HNE-AT complex were detected using plates coated with a polyclonal rabbit anti-human neutrophil elastase antibody (Sanquin, Amsterdam, the Netherlands). Bound complexes were detected by incubation with biotinylated monoclonal anti–α1-antitrypsin antibody followed by poly-horseradish peroxidase-labeled streptavidin. Results are expressed in ng/mL by reference to a standard curve of normal human citrated plasma in which HNE-AT complex were generated by incubating with purified elastase for 15 min at room temperature. The lower detection of the assay was 2 ng/mL. The coefficients of variation were 9.5% (inter-assay) and 5.7% (intra-assay).

Soluble p-selectin levels were assessed in plasma samples using commercially available ELISA (R&D MyBioSource, San Diego, California, USA). Lipoxin plasma levels were evaluated using a commercially available ELISA kit (Human lipoxin A4 (LXA4), Bio-Connect Services, Huissen, the Netherlands). All commercial ELISA's were performed according to the manufacturer's instructions. The coagulation potential in plasma was assessed using the calibrated automated thrombin generation (CAT) assay (Thrombinoscope BV). This method employs a low-affinity fluorogenic substrate for thrombin, and thereby enables continuous monitoring of thrombin activity in clotting plasma. For each measurement, 80 μL of human PPP was added to 20 μL of fluorogenic substrate, 20 μL of trigger reagent and calcium chloride, as previously reported (33). D-dimer fragments, a marker for fibrin formation and cleavage were determined as part of routine patient management using a latex-enhanced immuno assay (Innovance assay, Siemens Healthcare, Marburg, Germany).

### Statistical Analysis

Baseline characteristics were collected and differences between cohorts were analyzed using Chi-square testing. Differences in plasma levels of all markers between the DVT and PAD cohort were analyzed using the two samples *t*-test (parametric) or Mann-Whitney test (non-parametric). *T*-test results are shown as the mean and standard deviation, and Mann-Whitney results are shown as the median and 25th and 75th percentile. Subgroup analysis was performed with the one-way ANOVA (parametric) or the Kruskal-Wallis test (non-parametric). ANOVA results are shown as the mean and standard deviation, and Kruskal-Wallis results are shown as the median and 25th and 75th percentile. Correlations between d-dimer levels and markers of neutrophil activation were analyzed using linear regression. *P* < 0.05 were considered statistically significant. Identification and exclusion of outliers was performed using the ROUT method. Testing for normality was carried out with the D'Agostino Pearson test. All analyses were performed using GraphPad Prism version 7 for MAC OS X, GraphPad Software, La Jolla California USA, www.graphpad.com.

## Results

### Baseline Characteristics

Plasma from a total of 115 patients, 75 DVT patients and 40 PAD patients, was analyzed. Patient characteristics for these patients are shown in Table 1. Within the selected population of 75 patients from the DVT cohort we identified 10 patients with cardiovascular (CV) events, 2 of whom also had CV events prior to the DVT. These 10 patients were evenly distributed over the subgroups, with, respectively 3 patients in the DVT group without recurrence, 3 patients in the DVT group with recurrence, and 4 patients in the DVT group with PTS, leaving 65 DVT patients without CV events (Figure 1). Patients in the DVT cohort who experienced a CV event were older compared to patients in the other groups. PAD patients with CV events were more likely to be female. As expected, the use of oral anticoagulants was higher in DVT patients (63%) compared to PAD patients (0%), while the use of antiplatelet agents was much lower in DVT (5%) than in PAD (95%).

**Table 1:** Baseline characteristics, significant differences (*p* < 0.05) within cohorts depicted with^*^.

	**DVT**	**DVT**	**DVT**	**DVT**	**DVT**	**PAD**	**PAD**
	**No recurrence**	**Recurrence**	**PTS**	**CV event**	**No CV event**	**CV event**	**No CV event**
	***n* = 25**	***n* = 25**	***n* = 25**	***n* = 10**	***n* = 65**	***n* = 20**	***n* = 20**
Mean age (SD)	69.2 (31.4)	69.5 (11.8)	73.4 (12.9)	76 (7.5)^*^	69 (13.1)	68.7 (8.3)	68.3 (6)
Male gender (%)	21 (84)	15 (75)	9 (36)	7 (70)	42 (76.4)	9 (45)	15 (75)^*^
Anticoagulants (VKA, DOAC) (%)	17 (68)	19 (76)	11 (44)	7 (70)	40 (72.7)	0 (0)	0 (0)
Antiplatelet medication (%)	0 (0)	2 (8)	2 (8)	3 (30)^*^	1 (1.8)	20 (100)	18 (90)
Statin (%)	1 (4)	4 (16)	3 (12)	3 (30)^*^	5 (9.1)	14 (70)	15 (75)

*VKA, vitamin K antagonist; DOAC, direct oral anticoagulant*.

### Markers of Inflammation

**Figure 2:**
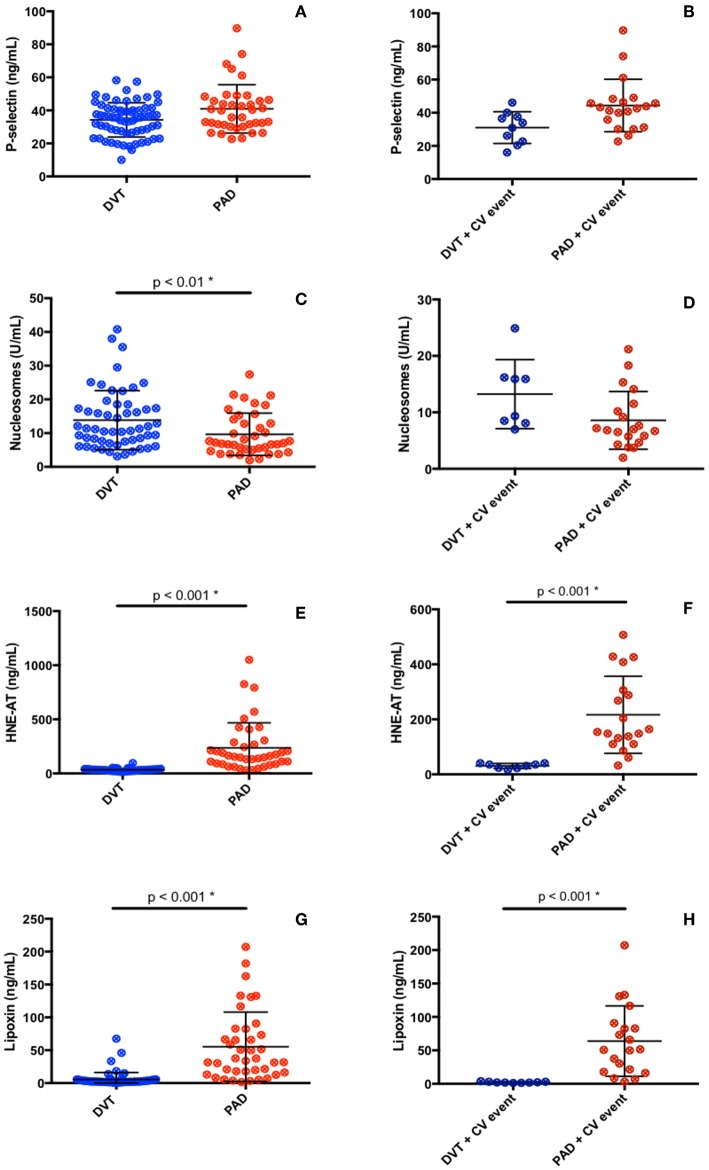
Inflammatory **(A–F)** and anti-inflammatory **(G, H)** markers in DVT patients and PAD patients.

Plasma levels of p-selectin were assessed to gather information on the activation of blood platelets and endothelial cells, with potential impact on recruitment of neutrophils. No difference in p-selectin levels in plasma from DVT and PAD patients was found (DVT 35.6 ng/mL (27.3–40.9), PAD 37.9 ng/mL (30.4–46.2), *p* = 0.08) (Figure 2A). P-selectin levels did not differ in DVT patients with CV events compared to PAD patients with events (Table 2, Figure 2B). P-selectin levels neither differed between the DVT subgroups (Table 3).

**Table 2:** Inflammatory markers in all DVT and PAD patients, and specifically in DVT and PAD patients with CV events.

	**All DVT**	**All PAD**	***P*-value**	**DVT + CV event**	**PAD + CV event**	***P*-value**
P-selectin (ng/mL)	35.6 (27.3–40.9)	37.9 (30.4–46.2)	NS	35.5 (25.3–40.4)	42.9 (32.3–47.8)	NS
Nucleosomes (U/mL)	11.3 (7.4–17.7)	7.1 (5.1–13.8)	< 0.01	12.6 (8.2–16.1)	6.9 (4.9–11.2)	NS
HNE-AT (ng/mL)	33.4 (23.5–40.5)	158 (88.1–283)	< 0.001	33.4 (23–39.4)	33.2 (22.9–38.1)	NS
Lipoxin A4 (ng/mL)	2.4 (1.7–4.8)	35.6 (16.6–80.1)	< 0.001	2 (1.5–3)	51.2 (18.7–88.7)	< 0.001

*P ≥ 0.05 are shown as non-significant (NS)*.

**Table 3:** Inflammatory markers in the DVT subgroups (a) no recurrence, (b) recurrence, and (c) PTS.

	**No recurrence**	**Recurrence**	***P*-value**	**PTS**	***P*-value**
**DVT SUBGROUPS**					
P-selectin (ng/mL)	30.4 (23–39.2)	36.4 (29.5–45.9)	NS	37 (27.8–42.9)	NS
Nucleosomes (U/mL)	8.7 (5.5–11.3)	11.3 (7.3–19.3)	NS	17 (15.3–24.9)	< 0.001
HNE-AT (ng/mL)	31.3 (20.6–35.9)	35.1 (24.5–38)	NS	40.4 (31.3–44.6)	0.046
Lipoxin A4 (ng/mL)	2.1 (1.6–4.3)	2.6 (1.8–4.2)	NS	3.4 (1.7–10.5)	NS

*P ≥ 0.05 are shown as non-significant (NS)*.

Neutrophil activation was assessed by measuring plasma nucleosome levels and levels of HNE-AT. Nucleosome plasma levels were significantly higher in the DVT cohort compared to the PAD cohort (11.3 U/mL (7.4–17.7) vs. 7.1 U/mL (5.1–13.8), *p* < 0.01) (Figure 2C). Plasma nucleosome levels in DVT patients with CV events were similar to nucleosomes levels in PAD patients with CV events [12.6 U/mL (8.2–16.1) vs. 6.9 U/mL (4.9–11.2), *p* = 0.32] (Figure 2D). Analysis of the DVT subgroups showed significantly higher nucleosome levels in patients with PTS (Table 3).

Plasma levels of HNE-AT were substantially lower in DVT patients compared to PAD patients (33.4 ng/mL (23.5–40.5) vs. 158 ng/mL (88.1–283), *p* < 0.001) (Figure 2E). DVT patients with CV events also had significantly lower HNE-AT levels compared to PAD patients with CV events (Table 2, Figure 2F). DVT subgroup analysis showed higher HNE-AT plasma levels in PTS patients (Table 3).

Anti-inflammatory marker lipoxin A4 was found to be significantly lower in DVT patients compared to PAD patients [2.4 ng/mL (1.7–4.8) vs. 35.6 ng/mL (16.6–80.1), *p* < 0.001] (Figure 2G). DVT patients with CV events had significantly lower lipoxin A4 levels compared to PAD patients with CV events (Table 2, Figure 2H). No differences were observed between DVT subgroups (Table 3).

**Figure 3:**
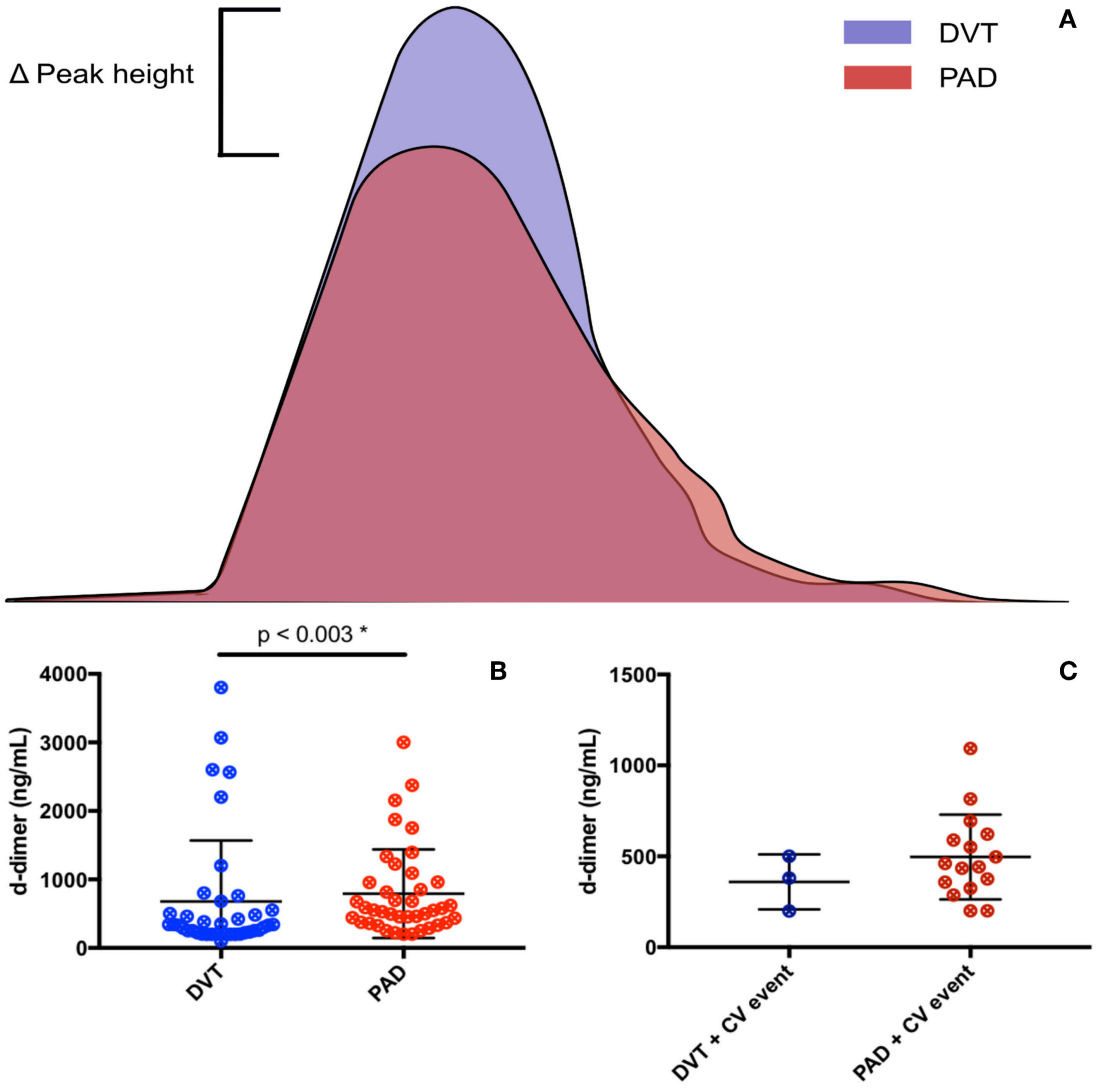
Coagulation markers in DVT patients and PAD patients. **(A)** Resembles thrombin generation curves of DVT and PAD patients, with an increased peak height seen in DVT patients. **(B)** Resembles d-dimer levels in DVT and PAD patients. **(C)** Resembles d-dimer levels in DVT and PAD patients with CV events.

The CAT thrombin generation assay yields data on lag time, endogenous thrombin potential (ETP) and peak height. Both the ETP and peak height are shown as “normalized” values. Thrombin generation and d-dimer levels were only assessed in patients not on anticoagulant treatment (Figure 3). The lag time was equal in DVT patients and PAD patients, with, respectively 5.5 min (4.8–6.7) and 5.5 min (4.7–6.2), *p* = 0.83. No difference in lag time was found between DVT patients with CV events and PAD patients with CV events (Table 4). Subgroup analysis of the DVT cohort showed no differences either (Table 5). We observed a higher ETP in DVT patients [160.4% (141.1–215.4)] compared to PAD patients [75.7% (59.3–93.1)], *p* < 0.001. A non-significant difference was found between DVT patients with CV events [144.7% (98.1–192.3)] and PAD patients with CV events [70.4 % (57.6–92.6)], *p* = 0.11. Subgroup analysis of the DVT cohort showed no differences in ETP (Table 5). Peak height was significantly higher in DVT patients compared to PAD patients [292.1% (177.9–330) vs. 82.2% (53.8–103.7), *p* < 0.001]. Higher peak height levels were also observed in DVT patients with CV events compared to PAD patients with CV events (Table 4). No differences in peak height were found between the DVT subgroups (Table 5).

**Table 4:** Coagulation markers in all DVT and PAD patients, and specifically in DVT and PAD patients with CV events.

	**All DVT**	**All PAD**	***P*-value**	**DVT + CV event**	**PAD + CV event**	***P*-value**
Lag time (min)	5.5 (4.7–6.2)	5.5 (4.8–6.7)	NS	5.8 (4.9–6.9)	5.5 (4.3–6.6)	NS
ETP (%)	160.4 (141.1–215.4)	75.5 (59.3–93.1)	< 0.001	144.7 (98.1–192.3)	70.4 (57.6–92.6)	NS
Peak height (%)	292.1 (177.9–330)	82.2 (53.8–103.7)	< 0.001	238 (169.3–319)	74.4 (51.4–100.9)	< 0.001
D-dimer (ng/mL)	330 (220–550)	550 (369–959)	0.003	380 (199–500)	524 (362.3–1024)	NS

*P ≥ 0.05 are shown as non-significant (NS)*.

**Table 5:** Coagulation markers in the DVT subgroups (a) no recurrence, (b) recurrence, and (c) PTS.

	**No recurrence**	**Recurrence**	***P*-value**	**PTS**	***P*-value**
**DVT SUBGROUPS**					
Lag time (min)	5.7 (4–6.7)	5.2 (4.8–6.7)	NS	6.5 (4.3–7.6)	NS
ETP (%)	151 (63.4–198.6)	183.2 (150.5–223.5)	NS	160.4 (111.5–203.6)	NS
Peak height (%)	236.2 (103.2–329.5)	295.1 (214.3–412.7)	NS	290.3 (173.6–330)	NS
D-dimer (ng/mL)	225 (199–297)	580 (330–1950)	0.008	380 (257–500)	0.001

*P ≥ 0.05 are shown as non-significant (NS)*.

D-dimer levels were significantly lower in the DVT cohort compared to the PAD cohort, respectively 330 ng/mL (220–550) and 550 ng/mL (369–959), *p* = 0.003. No differences in d-dimer levels were observed between DVT patients with CV events and PAD patients with CV events [380 ng/mL (199–500) vs. 524 ng/mL (362.3–1024), *p* = 0.99]. DVT subgroup analysis showed significantly lower d-dimer levels in DVT patients without recurrence compared to patients with recurrence and patients with PTS (Table 5). D-dimer levels in DVT patients did not correlate with nucleosome levels (*p* = 0.42) or HNE-AT levels (*p* = 0.14). There neither was a correlation with nucleosome levels (*p* = 0.49) nor HNE-AT levels (*p* = 0.38) in PAD patients.

## Discussion

**Figure 4:**
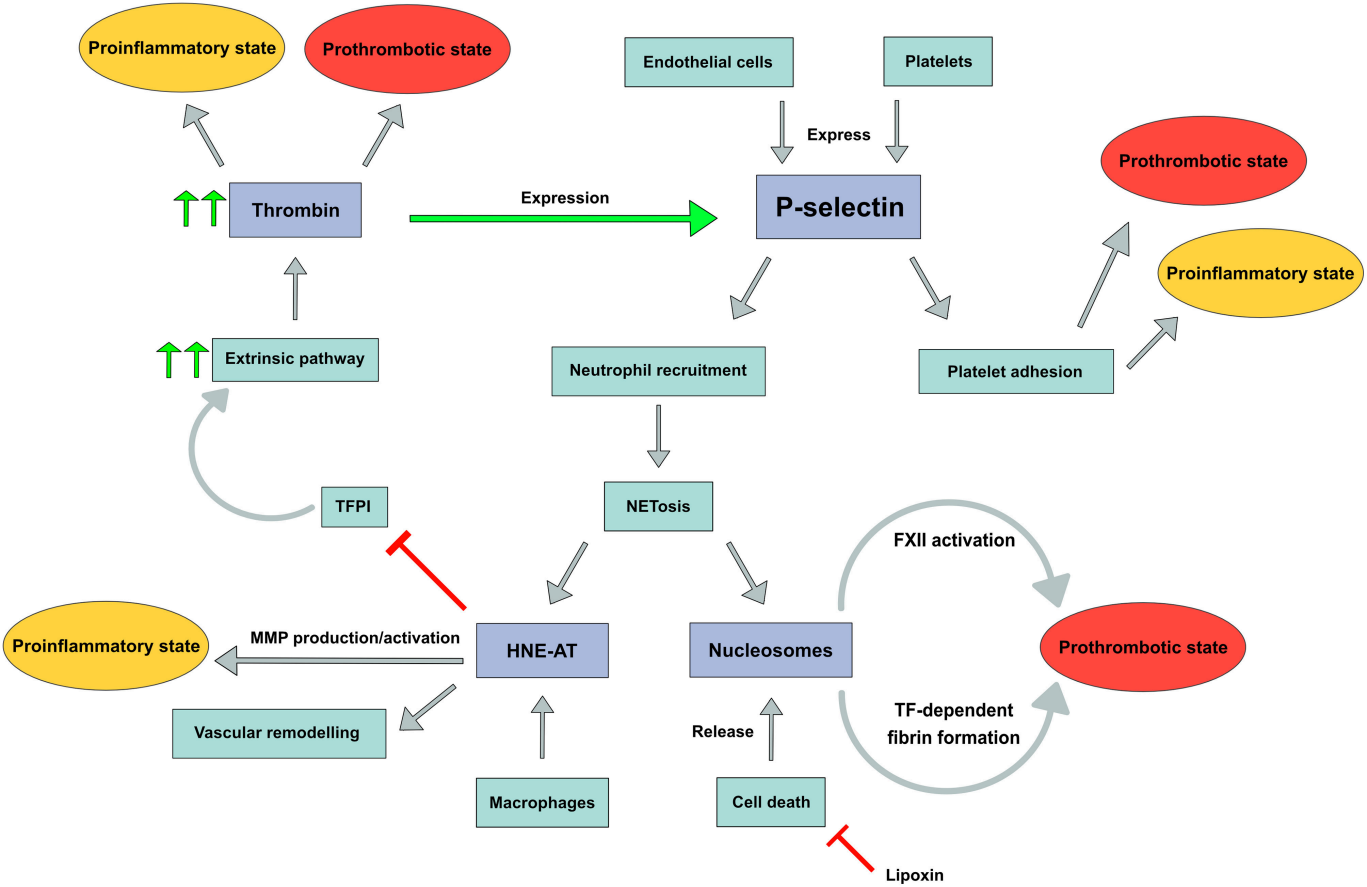
P-selectin expression with downstream pathways leading to a prothrombotic and/or proinflammatory state. FXII, Factor XII; TF, tissue factor; TFPI, tissue factor pathway inhibitor; MMP, matrix metalloproteinase.

In this explorative study we assessed the role of inflammation and coagulation in patients with non-acute DVT and patients with PAD in order to demonstrate a possible common biochemical background for the occurrence of atherothrombotic events in these patient populations. We used highly selected markers reflecting the interplay between platelet and endothelial activation (soluble p-selectin), neutrophil activation (nucleosomes and HNE-AT) as well as an inflammation inhibiting pathway component (lipoxin A4); Finally, we explored activity of the coagulation system by probing the potential to generate thrombin and by assessing a sensitive marker of coagulation activity, utilizing d-dimer (Figure 4).

We postulated that p-selectin would play a role in the interactions among platelets, leukocyte populations and endothelial cells, together with CD40 and several chemokines derived from platelets (34, 35). We demonstrate non-significantly higher levels of p-selectin in PAD patients compared to DVT patients, indicating that at least in the non-acute phase of vascular disease, there is no major difference in degree of platelet activation and to a lesser extent endothelial activation. This may be in part related to the fact that the clear majority of PAD patients were on antiplatelet therapy attenuating platelet reactivity to some extent (36).

In contrast to the lack of difference in p-selectin levels between patients with venous or arterial vascular disease, substantial differences in plasma levels of neutrophil products were observed. We demonstrate higher nucleosome levels in non-acute DVT patients, whereas higher HNE-AT levels were observed in PAD patients: the differences in plasma levels of both biomarkers were unexpected and may in part be explained by their specificity for NETosis. Activated neutrophils secrete nucleosomes, but apoptosis in any cell type will also give rise to increased nucleosome levels due to cell destruction (37). One could speculate that cell death, for instance due to venous congestion secondary to venous thrombosis, is a more prominent phenomenon in patients with a prior DVT than in those with arterial vascular disease. Subgroup analysis demonstrated different nucleosome levels within the DVT cohort, as DVT patients with PTS had higher nucleosome levels compared to DVT patients without PTS or recurrence. These results suggest more pronounced apoptosis with release of nucleosomes in relation to post-thrombotic vascular remodeling. In contrast, HNE-AT is a more specific marker for NETosis by neutrophils, although macrophages may also secrete HNE. Both neutrophils and macrophages are key players in atherosclerosis and atherothrombosis. Dollery et al. demonstrated the presence of HNE in macrophage-rich shoulders of atherosclerotic plaques but not in normal arteries. Moreover, these macrophages were shown to contain HNE mRNA. One may indeed expect that systemic HNE levels are also higher in PAD patients, reflecting the burden of atherosclerotic disease. It remains uncertain how substantial the contribution of neutrophil activation is toward the measured HNE-AT levels. While the association between nucleosomes and vascular remodeling has yet to be investigated, neutrophil elastase (measured as HNE-AT) is known to contribute to vascular remodeling and to promote plaque rupture (38). We did, however, not find an increase in CV events in patients with higher HNE-AT levels. Moreover, plasma levels of nucleosomes and HNE-AT in DVT patients with CV events differed significantly from plasma levels in PAD patients with these events, making it highly unlikely that neutrophil activation is a common mechanism of comparable significance in the occurrence of CV events in both cohorts.

A wide variety of mechanisms which counteract inflammatory processes have been identified in the past, and we aimed to assess anti-inflammatory capabilities by measuring lipoxin A4 levels. We demonstrate significantly higher lipoxin A4 levels in PAD patients in comparison to DVT patients. Lipoxin levels were not associated with the occurrence of CV events in both DVT and PAD patients. The relatively high levels of lipoxin A4 may at least in part be attributed to the actions of low dose aspirin, known to stimulate the levels of this anti-inflammatory mediator *in vivo* (39). Another factor may be the stronger activity of macrophages in patients with atherosclerosis, as compared to the DVT cohort, which may also modulate the production of lipoxin A4. Via this mechanism, a macrophage can limit its own apoptosis pathway, as previous studies have shown inhibition of apoptosis by lipoxin A4 (40).

## Neutrophils and hypercoagulability

We assessed hypercoagulability by measuring thrombin generation and d-dimer levels. Thrombin generation appeared to be increased in DVT patients, whereas d-dimer levels were higher in PAD patients. We found no difference in lag time, indicating that the onset of thrombin generation is essentially the same. ETP and peak height were, on the other hand, higher in DVT patients, implicating that a prethrombotic state is more pronounced in DVT patients. Due to low numbers, we were not able to show significant higher ETP values in DVT patients with CV events in comparison to PAD patients with CV events. These thrombin generation results are consistent with the concept that venous thrombosis is stronger dependent on blood coagulation reactivity, as compared to atherothrombotic events in atherosclerosis. A recent study pointed out that PAD patients have a “normal” thrombin generation profile compared to healthy controls, which is in line with our results. Kleinegris et al. however also demonstrated an increased ability to form stable clots in comparison healthy controls, mostly likely due to an increase in fibrinogen in atherosclerosis (28). These conclusions support our finding that d-dimer levels are increased in PAD patients. As more fibrin is formed, more fibrinolysis will likely occur, and thus more d-dimer will be formed.

Activated neutrophils can support coagulability via several mechanisms. In DVT, where nucleosomes are more pronounced, FXII can be auto-activated by negatively charged DNA backbones (41). Moreover, nucleosomes inhibit TFPI and thereby also promote coagulation (42). Human neutrophil elastase, increased in PAD, stimulates matrix metalloproteinase (MMP) production and activation. Furthermore, through inhibition of TFPI by elastase, thrombin and subsequent fibrin production as well as vascular remodeling may be promoted (16). Together, these data suggest different neutrophil (and probably macrophage) mediated mechanisms to be operational in venous and arterial vascular disease.

## Strengths and limitations

Thrombo-inflammation is involved in both arterial and venous thrombosis, and common pathophysiological pathways have yet to be elucidated. This study gives insights on the possible involvement of neutrophil activation on the occurrence of thrombotic events. Our results are hypothesis generating regarding pathogenic mechanisms in DVT and PAD.

The number of patients in the DVT and PAD cohort was too small to reliably analyse subgroups. Here, we also had to exclude DVT patients on anticoagulants to prevent interference with the outcome of thrombin generation testing and d-dimer levels. In general, the selected biomarkers are insufficient to document the contribution of platelets, neutrophils, and other relevant cells and microvesicles in these complex pathologies. In addition, even statistically significant differences for markers like nucleosomes should be considered with caution, given the lack of knowledge on the biological significance. For this matter, our data and interpretation of differences between populations, must be regarded as hypothesis generating.

## Conclusion

Our findings suggest that in patients with previous DVT neutrophil activity is not an important driver of CV events. In subjects with PAD, neutrophil activity is more pronounced and in part dampened by increased lipoxin A4. We did not detect any associations between neutrophil and nucleosome levels and markers of coagulation activity, suggesting that neutrophil activation is not a common driver of thrombosis risk in DVT and PAD.

## Author Contributions

AC-H, HC, HS, SZ, RO, and SB contributed to the conception and design of the study. AC-H, SB, and BK organized the database and performed the statistical analysis. BL performed the analysis of the immunoassay results. AC-H, HC, and BM performed the gathering of patients. BK wrote the first draft of the manuscript. All authors contributed to manuscript revision, read, and approved the submitted version.

### Conflict of Interest Statement

Current research from HS and HC is supported by grants from the Netherlands Heart Foundation (CVON RACE-5 and CONTRAST) and Bayer. HC is consultant for Stago and has received research support from Pfizer; advisory board functions for Pfizer, Bayer, JB Consulting. The remaining authors declare that the research was conducted in the absence of any commercial or financial relationships that could be construed as a potential conflict of interest.
